# Efficient Optimized YOLOv8 Model with Extended Vision

**DOI:** 10.3390/s24206506

**Published:** 2024-10-10

**Authors:** Qi Zhou, Zhou Wang, Yiwen Zhong, Fenglin Zhong, Lijin Wang

**Affiliations:** 1 College of Computer and Information Sciences, Fujian Agriculture and Forestry University, Fuzhou 350002, China; 5221139009@fafu.edu.cn (Q.Z.); 52311049016@fafu.edu.cn (Z.W.); yiwzhong@fafu.edu.cn (Y.Z.); 2 Key Laboratory of Smart Agriculture and Forestry, Fujian Province University, Fuzhou 350002, China; 3 College of Horticulture, Fujian Agriculture and Forestry University, Fuzhou 350002, China; zhong591@fafu.edu.cn; 4 Fujian Key Laboratory of Big Data Application and Intellectualization for Tea Industry, Wuyi University, Wuyishan 354300, China

**Keywords:** object detection, attention mechanism, complex environments, YOLOv8, efficient computing

## Abstract

In the field of object detection, enhancing algorithm performance in complex scenarios represents a fundamental technological challenge. To address this issue, this paper presents an efficient optimized YOLOv8 model with extended vision (YOLO-EV), which optimizes the performance of the YOLOv8 model through a series of innovative improvement measures and strategies. First, we propose a multi-branch group-enhanced fusion attention (MGEFA) module and integrate it into YOLO-EV, which significantly boosts the model’s feature extraction capabilities. Second, we enhance the existing spatial pyramid pooling fast (SPPF) layer by integrating large scale kernel attention (LSKA), improving the model’s efficiency in processing spatial information. Additionally, we replace the traditional IOU loss function with the Wise-IOU loss function, thereby enhancing localization accuracy across various target sizes. We also introduce a P6 layer to augment the model’s detection capabilities for multi-scale targets. Through network structure optimization, we achieve higher computational efficiency, ensuring that YOLO-EV consumes fewer computational resources than YOLOv8s. In the validation section, preliminary tests on the VOC12 dataset demonstrate YOLO-EV’s effectiveness in standard object detection tasks. Moreover, YOLO-EV has been applied to the CottonWeedDet12 and CropWeed datasets, which are characterized by complex scenes, diverse weed morphologies, significant occlusions, and numerous small targets. Experimental results indicate that YOLO-EV exhibits superior detection accuracy in these complex agricultural environments compared to the original YOLOv8s and other state-of-the-art models, effectively identifying and locating various types of weeds, thus demonstrating its significant practical application potential.

## 1. Introduction

Object detection, a critical branch of computer vision, has been driving technological innovation across various industries, including security surveillance, autonomous driving, industrial automation, and intelligent agriculture, since its inception [1]. Throughout the evolution of this technology, there has been a significant improvement in the accuracy and practicality of object detection methods, evolving from early feature-based approaches [2,3,4,5] to modern deep learning models [6,7,8,9,10,11]. In particular, the introduction of deep learning has greatly enhanced the performance of object detection algorithms in complex scenarios, enabling broader applications.

Currently, object detection extensively utilizes deep learning techniques, which are primarily categorized into single-stage and two-stage methods. Single-stage methods, originating with YOLO (You Only Look Once) [9], have evolved through iterations such as Single Shot MultiBox Detector (SSD) [10], YOLOv2 [12], RetinaNet [11], and YOLOv3 [13]. These methods process the entire image in a single forward pass to predict bounding boxes and class labels, known for their speed but often at the cost of accuracy. In contrast, two-stage methods began with R-CNN [6] and developed through techniques like spatial pyramid pooling [14] (SPPNet), Fast-RCNN [7], and Faster-RCNN [8], employing heuristic or CNN-based region proposal networks (RPNs) to generate region proposals followed by classification. Although these methods generally achieve higher accuracy, their computational redundancy significantly slows the inference process. Therefore, finding a balance between accuracy and speed is crucial.

Object detection technology has found extensive applications in various fields, notably particularly benefiting agriculture, especially in weed detection. Effective weed management is crucial to crop yield and quality, where traditional methods rely on labor-intensive, error-prone manual identification and mechanical removal [15]. With the rise of precision agriculture, using object detection to automatically identify and localize weeds has become an effective means to enhance agricultural productivity. High-resolution image capture and processing allow object detection models to accurately identify weed species and growth locations early, enabling more precise chemical or mechanical weed control, ultimately leading to cost savings, reduced chemical use, and environmental protection. In particular, the increasing demands in the agricultural field require researchers to develop efficient object detection methods that can adapt to complex natural environments. This makes the research in this paper not only theoretically significant but also highly promising in practical applications. YOLO-sesame, a model with enhanced accuracy for real-time weed detection in sesame fields, has been proposed [16], providing guidance in the detection of leaf diseases in sesame and other plant diseases. Similarly, a deep learning model based on YOLO v5 and attention mechanisms, specifically for real-time detection of invasive weed seedlings, has been developed [17]. Through multi-scale training and high-resolution image processing, the model’s performance has been improved, and field tests have shown its effectiveness in identifying target weeds. The performance of various versions of the YOLO object detector in detecting weeds in lawn environments has been evaluated, particularly YOLOv8, which has shown the best detection accuracy and speed [18]. Testing across multiple datasets has demonstrated the necessity of further model optimization in practical applications. Therefore, developing high-accuracy object detection methods for complex agricultural environments holds significant research value.

Existing models often perform poorly in complex agricultural scenes, especially in detecting small, densely distributed weeds [19]. Firstly, the high complexity of natural agricultural environments, including varying weed types, the similarity between crops and weeds, complex lighting conditions, and occlusions, poses challenges for accurate detection. Additionally, the diversity in the sizes, shapes, and colors of weeds and crops demands high generalizability and adaptability from detection models. These factors limit the effectiveness of traditional object detection models in agricultural weed detection, especially in handling small weeds and environments with high occlusion. To address the aforementioned issues, a strategy to expand the model’s vision has been proposed, which involves enhancing the model’s feature extraction and detection capabilities to improve its adaptability to complex environments. Specifically, this paper introduces a series of innovative techniques aimed at improving the model’s performance in multi-scale information processing through more efficient feature fusion and attention mechanisms.

By introducing the concept of extended vision, the innovation in this paper goes beyond simple model iteration, significantly enhancing the model’s perception capabilities in complex environments on a broader scale. Specifically, we introduce the YOLO-EV model, initially validated on the VOC12 dataset and demonstrating effective agricultural weed detection. This model integrates innovative technologies, including multi-branch group-enhanced fusion attention (MGEFA) and large-scale kernel attention (LSKA) [20], and incorporates a P6 layer along with Wise-IOU [21], enriching the model’s field of view and improving accuracy and efficiency in challenging environments. The concept of ‘extended vision’ in YOLO-EV refers to its enhanced capability to perceive and analyze a wider range of spatial and contextual details within complex scenes. Specifically, ‘extended vision’ is achieved through the integration of MGEFA, which boosts feature extraction by handling multiple scales of input simultaneously, and LSKA, which focuses on improving the granularity of the attention mechanism, thus allowing finer control over which parts of an image are emphasized during the detection process. Additionally, the incorporation of the P6 layer extends the model’s ability to process multi-scale features, which is crucial for detecting objects of different scales and effectively handling occlusions, thereby significantly broadening the application scope of YOLO-EV in various agricultural settings.

Through a detailed analysis of complex agricultural scenarios, this paper proposes specific technical solutions, demonstrating their feasibility and significance in a wide range of practical applications. In particular, innovations such as the multi-branch group-enhanced fusion attention mechanism are introduced to address the limitations of existing technologies, effectively tackling object detection challenges in complex environments. This research not only improves the accuracy and efficiency of object detection technology but also provides practical guidance for fields like precision agriculture. The main innovations and contributions of this study are as follows:

(1) We propose an advanced attention mechanism, optimizing feature extraction through multi-branch and group convolution techniques, significantly enhancing model detection precision and robustness, and demonstrating superior performance in capturing multi-scale information and details.
(2) We propose the YOLO-EV model, employing enhanced feature fusion and attention techniques, surpassing YOLOv8 and a series of classic models in terms of accuracy and efficiency.
(3) We validate the real-time, high-accuracy detection capability of YOLO-EV across multiple datasets, highlighting its adaptability and effectiveness in general situations and various complex agricultural environments.

Overall, the research in this paper not only holds significant application value for agricultural technology but also offers new insights and technical support for object detection challenges in other complex environments, thus carrying important innovative significance in the field of object detection.

## 2. Materials and Methods

### 2.1. Technical Background Introduction

Object detection is a critical task in computer vision, aimed at identifying object categories within an image and determining their locations. With the rapid advancement of deep learning, object detection technologies have made significant progress but still face challenges in complex scenes. This has driven researchers to explore more advanced technologies such as attention mechanisms [22,23,24], multi-scale feature enhancement, and improved IoU loss functions to boost model performance.

Attention mechanisms play a key role in enhancing the performance of deep learning models. They mimic the human visual attention system by focusing computational resources on important regions while ignoring irrelevant information. In computer vision, attention mechanisms are widely applied in tasks like image classification, object detection, and semantic segmentation. Typical attention mechanisms include SE-Net [22], which models inter-channel relationships to enhance feature representation; CBAM [23], which combines spatial and channel attentions, further improving the model’s perception of important areas; and ECA [24], which maintains computational efficiency while enhancing attention effects. Introducing attention mechanisms helps models more accurately capture critical features and enhances adaptability to complex scenes.

Multi-scale feature enhancement is another crucial strategy for improving object detection performance. Due to the diversity in object sizes and shapes, single-scale features often fail to comprehensively describe target information. FPN [25] enhances multi-scale information integration through a top-down feature fusion, improving the detection capability for objects of various sizes, particularly small ones. PANet [26] builds on this by adding a bottom-up path, further strengthening feature fusion and enabling powerful feature expression across different scales. These methods significantly enhance model performance on targets of various sizes.

Intersection over Union (IoU) is a key metric for evaluating the overlap between predicted and actual bounding boxes. Traditional L1 and L2 loss functions do not fully reflect differences in the position and shape of bounding boxes, limiting model precision. Thus, IoU-based loss functions like IoU loss [27], GIoU [28], DIoU [29], and CIoU [30] have been proposed, using IoU as a metric and considering overlap, center distance, and shape differences. These improved loss functions more effectively guide model training, enhancing target localization accuracy. Recent methods such as Wise-IoU [21] further optimize IoU loss calculations, improving model convergence speed and generalization capability.

In summary, to address the challenges of object detection in complex scenarios, integrating advanced attention mechanisms, multi-scale feature enhancement, and improved IoU loss functions is an effective strategy. The combined application of these technologies not only enhances model detection performance but also provides technical support for practical applications in fields like agriculture, significantly improving productivity and quality.

### 2.2. Dataset

**Figure 1:**
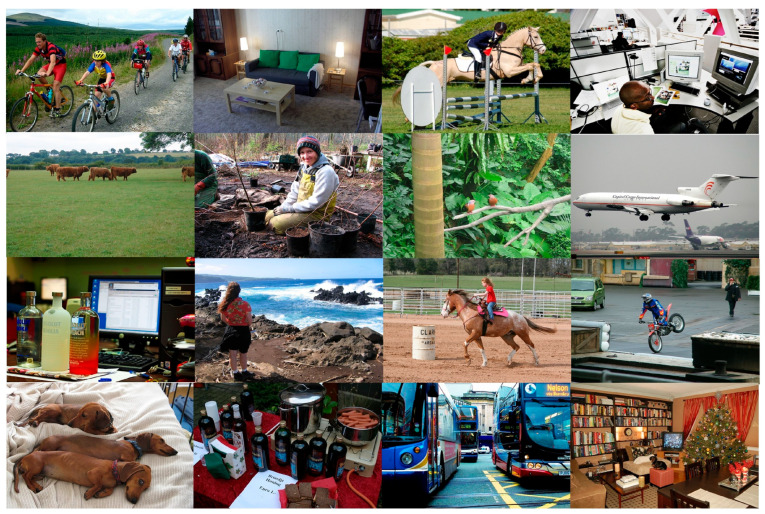
The images from the VOC2012 dataset.

To comprehensively evaluate the performance and application potential of the YOLO-EV model, this study employed three publicly available datasets for detailed experimental validation. Initially, preliminary testing was conducted on the PASCAL VOC 2012 (VOC12) dataset [31]. The VOC12 dataset is a widely used benchmark in computer vision, covering a variety of classification and object detection tasks. With its rich image content comprising objects from 20 categories, it provides an initial verification of the model’s general performance. Subsequently, to further test the model’s effectiveness in specific agricultural scenarios, the CottonWeedDet12 (CWD12) dataset [32] was utilized. This dataset is specifically designed for weed detection in cotton fields, featuring complex backgrounds and diverse weed species, thereby specifically testing the model’s capability in handling agriculture-related tasks. Finally, the model was also evaluated on the CropWeed dataset, which is tailored to assess the ability to distinguish between crops and weeds, highlighting the model’s recognition capabilities for subtle visual differences between crops and weeds. Systematic evaluations on these datasets allow for a comprehensive understanding of the proposed model’s performance in both general object detection and specific agricultural applications. Figure 1 showcases the VOC12 dataset, which includes a rich variety of scenes and categories.

### 2.3. Data Augmentation

**Figure 2:**
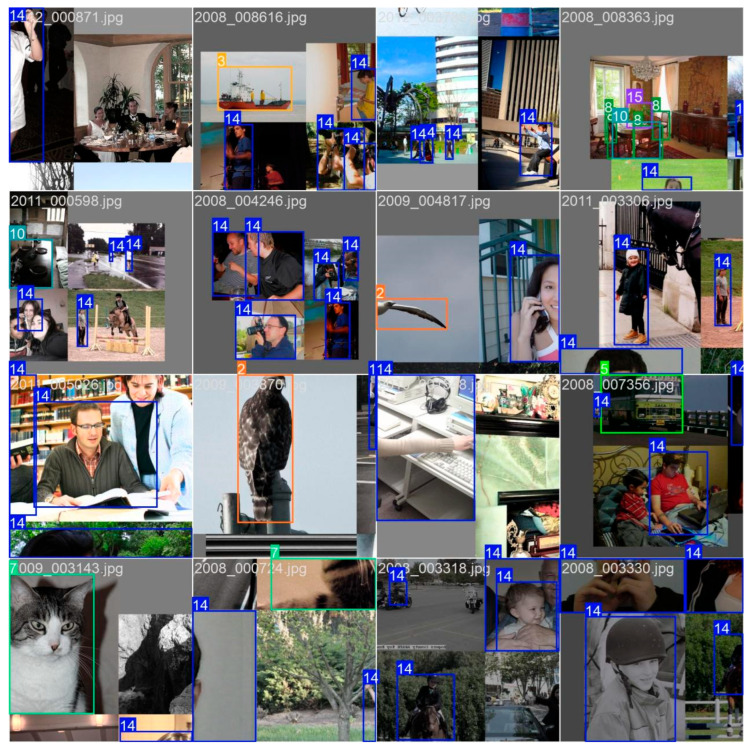
Visualization of data augmentation during training.

In this study, data augmentation techniques were employed to enhance the model’s generalization capabilities and prevent overfitting, which are crucial aspects of the training process for the YOLOv8 [24] and YOLO-EV models. Data augmentation not only artificially expands the training dataset but also enhances the model’s robustness when encountering unseen data. Specifically, the following data augmentation methods were utilized: random cropping, scaling, flipping, and color adjustment. Random cropping helps the model learn to recognize objects from partial views; scaling trains the model to identify targets of various sizes; flipping (both horizontal and vertical) increases the diversity of the scenes, enabling the model to adapt to different orientations of objects within images; color adjustments (such as changes in brightness, contrast, saturation, and hue) simulate visual effects under varying lighting conditions, enhancing the model’s adaptability to changes in illumination. The combined application of these methods significantly enriches the diversity of the training data, contributing to improved performance and reliability of the model in practical applications. Training the model under different environments and conditions ensures higher accuracy and stronger adaptability in actual operations. Figure 2 visualizes the data augmentation processes used during training.

### 2.4. YOLOv8

**Figure 3:**
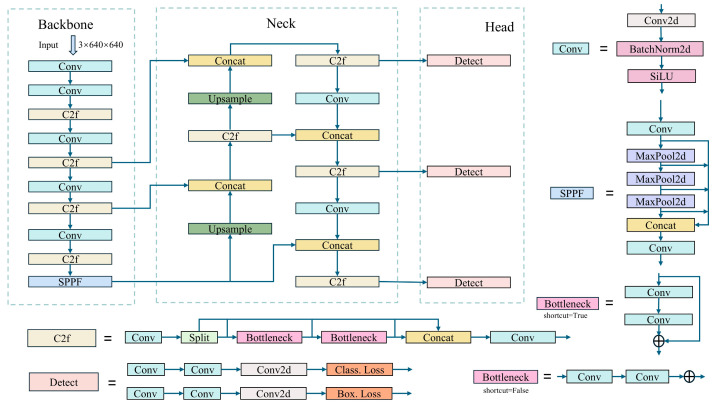
YOLOv8 model architecture.

YOLOv8 is an outstanding new version in the YOLO series of algorithms [9,12,13,33,34,35,36,37], which has optimized performance and precision while inheriting the high-speed processing capabilities of its predecessors. Figure 3 displays the architectural diagram of YOLOv8. The design of YOLOv8 embodies several innovative features. Firstly, it employs a deeper and more complex neural network to support more refined feature learning. Additionally, YOLOv8 introduces the latest convolutional techniques in its feature extractor, such as multi-scale convolution and more effective activation functions, which help the model to better capture detailed information in images. Furthermore, YOLOv8 optimizes its loss function design by introducing a new method for calculating localization loss, thereby improving accuracy in detecting objects of various sizes. YOLOv8 also enhances its adaptability by dynamically adjusting the network structure to accommodate different input sizes, a feature that allows YOLOv8 to maintain efficient performance and detection capabilities across various devices. To adapt to different application needs, YOLOv8 supports multiple training and inference modes, enabling it to operate efficiently even in resource-constrained environments. Thus, YOLOv8 not only maintains the speed and accuracy advantages of the YOLO series but also significantly improves in terms of model applicability and robustness, making it a highly competitive tool in the field of object detection. Despite its impressive performance, YOLOv8 still faces challenges in handling scenes with dense, occluded, or small objects. Particularly in environments where objects overlap or are partially obscured, the model struggles to capture sufficient feature information, resulting in decreased recognition accuracy. Additionally, the detection of small objects is challenging due to their limited features. Although YOLOv8 attempts to address these issues through the introduction of multi-scale convolution and dynamic network structural adjustments, optimizing algorithm performance for these scenarios remains a key direction for further enhancement.

### 2.5. Innovative Network Design: YOLO-EV

YOLOv8 inherits and advances a series of advantages from YOLOv5, while introducing multiple technical innovations to enhance the model’s accuracy, speed, and adaptability to various complex scenarios. Compared to YOLOv5, YOLOv8 has undergone significant improvements in network architecture, including the substitution of the C3 module with a more efficient feature fusion technology, C2f, optimized anchor strategies, and the use of decoupled heads in place of coupled heads. Additionally, YOLOv8 incorporates support for the latest advancements in deep learning algorithms, such as more advanced activation functions and batch normalization processes, which contribute to improved training stability and generalization capabilities.

**Figure 4:**
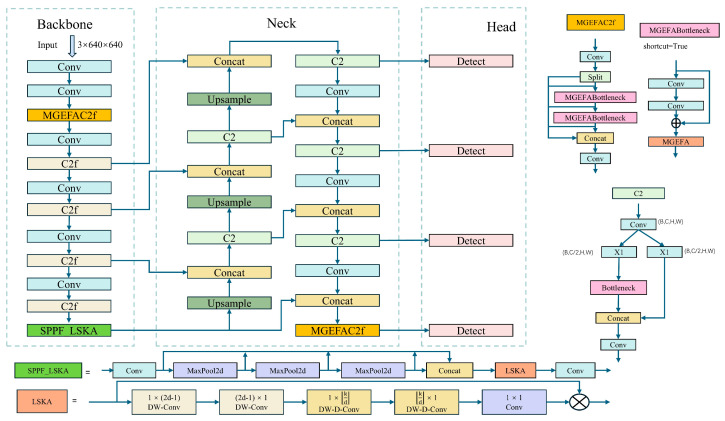
YOLO-EV model architecture. Compared to YOLOv8, YOLO-EV incorporates the innovative MGEFAC2f module, SPPF_LSKA, and an additional P6 layer, resulting in an extra detection head. Moreover, it replaces the traditional IoU metric with Wise-IoU. The specific implementation details will be elaborated in the following sections.

Despite these advancements, YOLOv8 still faces limitations in object detection tasks under extreme conditions. For instance, in environments with high occlusion or significant changes in lighting, YOLOv8′s performance may still be affected [38]. Furthermore, although there has been an increase in both speed and accuracy, the complexity of YOLOv8 may lead to reduced efficiency in practical applications on resource-limited devices [39]. In response to these challenges, we propose a new object detection network, YOLO-EV, as shown in Figure 4, with the following main innovations:

(1) To enhance the model’s feature extraction capabilities, we incorporate the proposed multi-branch group-enhanced fusion attention mechanism (MGEFA) into the backbone network of YOLOv8, significantly boosting its feature extraction and information integration capabilities and thereby improving detection accuracy and computational efficiency.
(2) In order to improve the accuracy and adaptability of object detection, we enhance the SPPF in YOLOv8 using LSKA and propose the SPPF_LSKA module to improve computational efficiency and model robustness.
(3) To address the challenges of detecting targets of varying scales in complex environments, we add a P6 layer to YOLOv8, enhancing detection precision and robustness. Furthermore, we replace the C2f module with the C2 module in the neck network to reduce computational demand and increase inference speed.
(4) To further enhance the model’s detection accuracy, we use WiseIoU in place of CIoU, enhancing training stability and robustness and accelerating model convergence.

As depicted in Figure 4, the network model comprises three main parts: the backbone, neck, and head. The backbone network is crucial for extracting network features, and the quality of feature extraction significantly impacts model performance. Therefore, the backbone network incorporates MGEFA to enhance feature extraction capabilities, helping to extract more comprehensive information. To address the challenges of varying target scales in complex environments, a P6 layer is added to the backbone network, improving detection accuracy and robustness. LSKA extends the SPPF module to further enhance feature extraction and information integration capabilities, improving YOLOv8′s object detection accuracy and robustness in multi-scale and complex backgrounds.

The neck network further integrates the features from different scales produced by the backbone network. The introduction of the P6 layer and MGEFA enables the neck network to integrate information from four different scales. The head network employs a decoupled approach for organizing and integrating information. Moreover, WiseIou is used to enhance sensitivity to objects, improving detection accuracy.

Overall, the model significantly enhances the recognition capabilities for small targets and targets in complex backgrounds through the introduction of new visual attention mechanisms and more flexible feature fusion strategies. Additionally, by optimizing the computational process and reducing unnecessary parameters, the model minimally increases the computational demand without sacrificing detection accuracy, making it more suitable for operation on edge devices. These advantages make the YOLO-EV model not only superior in traditional detection tasks but also highly practical in real-world applications. The following sections will provide a detailed introduction to the improved modules.

#### 2.5.1. Multi-Branch Group-Enhanced Fusion Attention (MGEFA)

**Figure 5:**
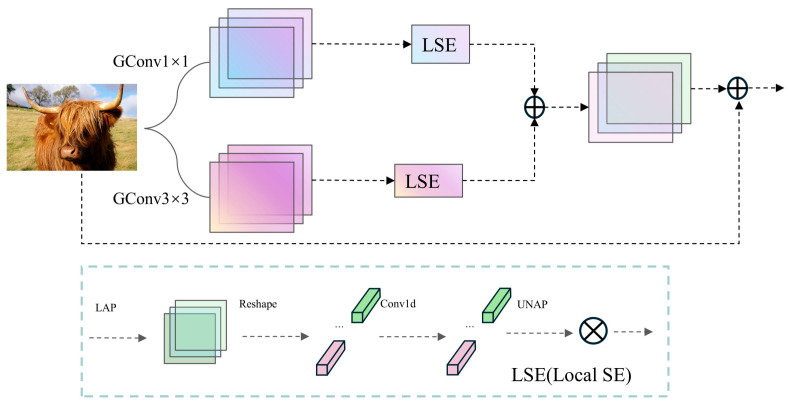
MGEFA network architecture.

The multi-branch group-enhanced fusion attention mechanism (MGEFA) is a method that enhances feature extraction and fusion capabilities through multiple branches and group convolutions, hence its name. Its specific implementation is illustrated in Figure 5.

The core concept of the MGEFA mechanism lies in using multiple branches to extract features and integrating spatial and channel information through group convolutions and localized spatial channel attention (LSE) [40]. Initially, multiple branches are constructed using convolution kernels of different sizes (e.g., 1 × 1 and 3 × 3 kernels), with each branch independently performing feature extraction. This multi-branch design captures features at various scales, thereby enhancing the model’s detection capabilities for targets of different sizes and shapes. After convolution, features are normalized and transformed non-linearly using batch normalization [41] and ReLU activation functions [42] to ensure stability and expressive power in subsequent processing. Subsequently, MGEFA employs localized spatial channel attention (LSE) to further process features. LSE reduces each branch’s feature map to a local area through adaptive average pooling and extracts local channel attention information using one-dimensional convolution. Specifically, local features undergo shape transformation, local attention is computed via one-dimensional convolution, and then the dimensions are restored to the original feature map size. By fusing local and global information, the model can more accurately capture spatial and channel information in images. Finally, MGEFA integrates the features from each branch with the original input features through a residual structure, forming the final output features. Residual connections not only alleviate the problem of gradient vanishing but also preserve key information from the input features, making the fused features richer and more varied. Through the comprehensive application of multi-branch feature extraction, group convolutions, and attention mechanisms, MGEFA effectively enhances the model’s feature representation capabilities and detection accuracy. Overall, the MGEFA mechanism demonstrates significant advantages in multi-scale feature extraction, information integration, and feature fusion, providing robust support for the performance of YOLO-EV.

**Figure 6:**
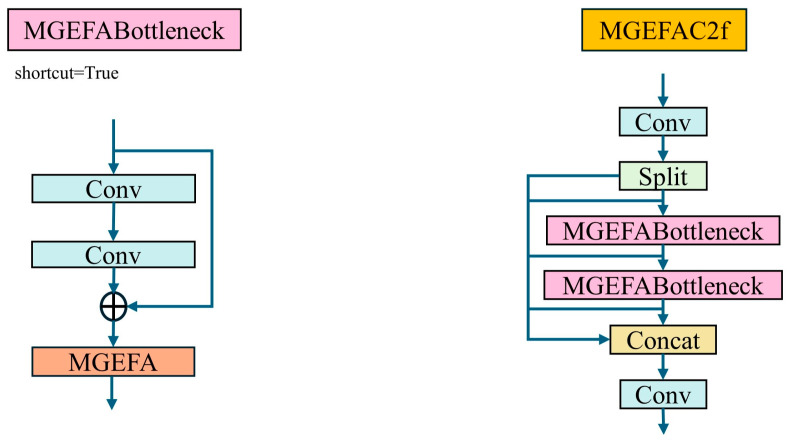
MGEFAC2f module structure.

To seamlessly integrate MGEFA into YOLO-EV, we propose MGEFAC2f, which combines MGEFA with C2f, as shown in Figure 6. This integration method combines the multi-branch feature extraction and attention mechanisms of MGEFA with the cascaded convolutional feature extraction strategy of C2f.

C2f operates by cascading multiple convolutional layers, allowing each layer to capture the features from the previous layer and subsequently extract richer feature information. By integrating C2f with MGEFA, the model can perform feature extraction and fusion across multiple scales and layers, thereby enhancing the expressiveness of features and the detection accuracy of the model. Specifically, MGEFAC2f first processes these features through the C2f module, followed by multi-branch feature extraction and fusion using the MGEFA module, which enhances each layer’s features based on those of the preceding layer. This design not only captures more detailed information but also improves the global representational ability of features, thus enhancing the model’s robustness and accuracy in complex environments. By integrating MGEFA and C2f, MGEFAC2f offers significant advantages in multi-scale feature extraction, information fusion, and feature enhancement, providing stronger support for the detection performance of YOLO-EV.

#### 2.5.2. Large Scale Kernel Attention (LSKA)

LSKA is an innovative visual attention mechanism that significantly reduces computational complexity and memory usage by redesigning traditional large kernel attention (LKA), thereby enhancing the efficiency and accuracy of object detection models.

The core idea of LSKA is to decompose the traditional 2D convolutional kernel into two sequential 1D kernels (horizontal and vertical), which effectively reduces the number of parameters and computational costs. Specifically, through this horizontal and vertical kernel decomposition, LSKA achieves depthwise separable convolution with very large kernel sizes, thus avoiding the high computational complexity and memory demands associated with traditional depthwise convolution when dealing with large-sized kernels. This innovative design allows LSKA to maintain performance comparable to standard LKA while significantly reducing computational complexity and memory consumption, enhancing the model’s operational efficiency. When applied within a visual attention network (VAN) [43], LSKA demonstrates outstanding processing capabilities, especially in complex scenarios requiring the recognition of long-range dependencies within images. By effectively capturing long-distance information, the model can more accurately identify and locate objects within images, particularly excelling in specific tasks like the detection of varied shapes and sizes of agricultural weeds.

**Figure 7:**
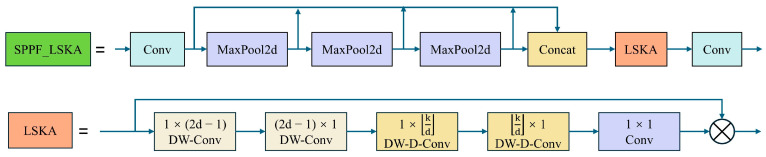
LSKA module and SPPF_LSKA module.

To address the limitations of the original SPPF (spatial pyramid pooling fast) in handling spatial information in complex scenes, this paper introduces the SPPF_LSKA module, which integrates LSKA, as shown in Figure 7. Although the traditional SPPF module can handle multi-scale objects to a certain extent, its capability to capture spatial information remains insufficient in highly complex backgrounds, limiting the overall recognition accuracy and speed of the model. By incorporating LSKA, the SPPF_LSKA module not only significantly enhances the processing of spatial information but also improves the balance between detection accuracy and processing speed. Test results on multiple standard datasets indicate that the introduction of LSKA effectively enhances the overall performance of the model, particularly its recognition capabilities in highly complex scenes.

#### 2.5.3. Multi-Scale Feature Enhancement (P6)

This section introduces the newly incorporated P6 layer in the YOLO-EV model. The primary intent of this layer’s design is to allow the model to capture richer information and a broader field of view, especially to enhance detection capabilities for objects of varying sizes and objects in occluded conditions. This design holds significant practical relevance for object detection in complex scenarios, notably in applications such as agricultural monitoring, urban surveillance, and monitoring in natural environments.

The P6 layer is essentially an expansion based on the traditional feature pyramid network (FPN) [25]. In a standard FPN, different scale feature maps are fused to enhance the model’s ability to recognize targets of varying sizes. The P6 layer is specifically added to process larger scale image features, thereby enabling the model to respond better to large targets. This design allows the YOLO-EV model not only to effectively handle small or medium-sized targets but also to more accurately recognize and locate large targets. Technically, the P6 layer achieves this by further downsampling and merging higher-level features. During this process, through appropriate convolution and downsampling operations, the P6 layer extracts crucial information from deeper network layers and combines it with high-level features in the existing feature pyramid. This structural design not only enhances the model’s perception of large targets but also improves performance in occluded scenarios.

Additionally, within the neck network that includes P6, the C2f module has been replaced with C2. In deeper layers of the feature pyramid like P6, feature maps have relatively lower resolution but more channels. At this stage, using the C2 module reduces computational load and improves inference speed while also decreasing memory consumption, which can be crucial in practical applications. By replacing C2f with the simpler C2, feature distortion due to excessive fusion can be avoided, thus enhancing model stability. Moreover, the structure of the C2 module is simpler, meaning that during the model training process, gradient propagation is more direct, aiding the model in finding optimal solutions more quickly. Furthermore, the introduction of the P6 layer also optimizes the model’s adaptability to complex backgrounds and environmental changes. In applications such as agricultural monitoring or natural environment surveillance, backgrounds are often highly complex, and targets may be severely occluded. The P6 layer enables the model to better separate targets from these complex environments, enhancing overall detection accuracy and robustness.

Experimental results show that the YOLO-EV model with the P6 layer has significantly improved performance in handling large-sized targets and complex occlusion scenarios. This improvement not only enhances the model’s practicality but also provides strong technical support for future object detection in more challenging environments.

#### 2.5.4. WISE-IoU (WIoU)

This section introduces an improved Intersection over Union (IoU) loss function used in the YOLO-EV model, named Wise-IoU (WIoU). WIoU enhances the model’s accuracy in target localization, particularly when dealing with targets that are occluded or vary significantly in size, by incorporating a dynamic non-monotonic focusing mechanism.

The core concept of Wise-IoU is to dynamically adjust the gradient gain for each anchor box by estimating its outlier degree, allowing the loss function to focus not only on high-quality anchor boxes but also appropriately address medium or low-quality ones. This method, compared to traditional IoU loss functions, more intelligently allocates gradients, reducing the negative impact caused by low-quality anchor boxes, thereby overall enhancing the performance of the detector. In implementation, WIoU first defines a loss function based on IoU and then introduces a dynamic function to adjust the gradient gain for each anchor box. This adjustment is based on the relationship between the anchor box’s IoU value and a preset threshold. When an anchor box’s IoU value is close to this threshold, it receives a higher gradient gain; as the IoU value moves away from the threshold, the gradient gain gradually decreases. This non-monotonic gradient adjustment strategy allows the model to focus more on those cases with moderate quality but high potential for model improvement during training.

Moreover, WIoU optimizes the processing of anchor boxes through an intelligent gradient gain allocation strategy, which uses outlier degrees rather than simple IoU values for anchor box quality assessment. This enables WIoU to more accurately assess the quality of an anchor box and dynamically adjust the gradient gain based on the specific circumstances of the anchor box, improving the model’s ability to locate targets in complex scenes. Experimental results show that WIoU outperforms traditional IoU and other related variants in applications across multiple standard datasets, confirming its effectiveness and robustness in practical applications.

### 2.6. Model Evaluation Metrics

This section provides a comprehensive overview of the various metrics used to evaluate the performance of the YOLO-EV model. These metrics include traditional ones such as accuracy, precision, recall, average precision (AP), and mean average precision (mAP), as well as more rigorous criteria like mAP@0.5:0.95.

Accuracy is an intuitive metric that measures the overall correctness of the model’s predictions by calculating the proportion of samples correctly predicted in the dataset. This metric reflects the model’s overall ability to correctly predict both positive and negative samples. Precision is a metric used to assess the accuracy of the model in predicting positive samples. It is calculated as the ratio of true positives (TP) to the total number of samples predicted as positive by the model (TP + false positives (FP)), as shown in Formula (1).

(1) $$Precision(P)=\frac{TP}{TP+FP}$$

Recall, also known as sensitivity or the true positive rate, is used to measure the proportion of actual positive samples correctly identified by the model from all actual positives. It is an important metric for assessing the model’s capability to capture positive samples, as shown in Formula (2).

(2) $$Recall(R)=\frac{TP}{TP+FN}$$

Average precision (AP) primarily reflects the model’s ability to balance precision and recall across different categories of targets. It is obtained by calculating the area under the precision-recall curve at different confidence thresholds, as shown in Formula (3).

(3) $$AP=\int_{0}^{1}P\left(R\right)dR$$

Mean average precision (mAP) is a widely used performance metric in the field of object detection, based on precision and recall at different confidence thresholds. mAP is calculated by averaging the average precision (AP) for each category and then taking the average of the APs across all categories, thus providing an overall performance evaluation for multi-category detection tasks. As shown in Formula (4), the symbol i represents a specific category, while AP(i) represents the average precision corresponding to that category and n represents the total number of categories. This paper primarily utilizes mAP@0.5, which is the mAP at an Intersection over Union (IoU) threshold of 0.5, one of the most commonly used standards in evaluating object detection algorithms.

(4) $$mAP=\frac{1}{n}\sum_{i=1}^{n}AP(i)$$

Furthermore, mAP@0.5:0.95 is a more stringent evaluation criterion, which calculates the average mAP from IoU thresholds ranging from 0.50 to 0.95, with increments of 0.05. This calculation method more comprehensively considers the model’s performance under different overlap requirements, providing a more accurate reflection of the model’s ability to precisely identify target locations. In this paper, in addition to using mAP@0.5 as the primary evaluation standard, we also reference mAP@0.5:0.95 to comprehensively assess the model’s performance in various complex scenarios, ensuring that the proposed improvements maintain efficiency and accuracy under a broad range of application conditions.

In assessing the model’s efficiency and speed, metrics such as floating point operations (FLOPs), the number of model parameters, and frames per second (FPS) are also utilized. These metrics not only evaluate the computational complexity and resource consumption of the model but also measure the model’s inference speed in practical applications. Through these multidimensional evaluation metrics, the performance and practicality of the YOLO-EV model in handling complex object detection tasks can be comprehensively assessed and validated.

## 3. Experimental Setup and Discussion

### 3.1. Experimental Configuration

The experimental setup used in this study, including the hardware platform and software environment, is detailed in Table 1.

Regarding model training configurations, the SGD optimizer was selected to optimize the model’s performance on the dataset, with an initial learning rate set at 0.01 to quickly approach the optimal solution. The training process was set to a maximum of 300 epochs. In the VOC dataset, 40 images per batch were processed (batch size = 40), and in the CWD12 and CropWeed datasets, 16 images per batch were processed (batch size = 16). All other parameters were consistent with the official source of the code. These experimental settings not only ensured the efficiency and uniformity of the training process, enhancing the persuasiveness of all experimental results, but also provided a solid foundation for the evaluation of the model.

### 3.2. Ablation Study

In this section, ablation experiments were conducted to validate the roles of MGEFA, the P6 layer, and LSKA in enhancing the performance of the YOLOv8 model. By systematically removing or replacing these technical components, their individual contributions to the model’s detection accuracy in complex scenarios were assessed. All models, except for the baseline YOLOv8s, incorporated Wise-IoU, and the experimental results in Table 2 confirm that Wise-IoU enhances model performance without additional computational cost. Specifically, MGEFA significantly improved model detection accuracy by enhancing feature extraction and fusion capabilities; the introduction of the P6 layer optimized the model’s ability to handle large targets and complex backgrounds; and LSKA, by reducing computational complexity and enhancing spatial information processing efficiency, further strengthened the model’s capability to capture details. The results of the ablation studies show that the comprehensive application of these techniques significantly enhanced the YOLOv8 model’s performance in object detection, demonstrating their effectiveness and applicability in real-world scenarios. The results of the ablation study are presented in Table 2.

**Figure 8:**
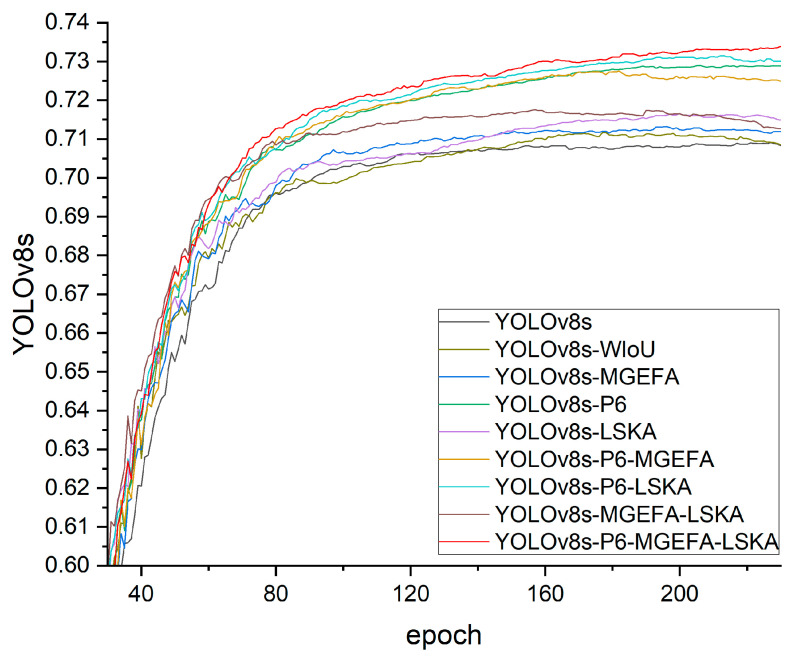
Comparison of ablation study results.

As shown in Table 2, each component contributed differently to the overall performance enhancement. The introduction of MGEFA led to a noticeable increase in mAP@0.5, which can be attributed to its ability to better capture multi-scale features and improve the fusion of contextual information. This enhancement is particularly significant in complex environments where objects vary in size and appearance. Figure 8 displays the training convergence curves of various models in the ablation study, showing that they had essentially converged by 200 epochs, confirming that 300 epochs are sufficient.

By systematically removing or replacing specific technological components, we conducted a detailed analysis of their individual and combined contributions to model performance. The experimental results demonstrated that the baseline model, YOLOv8s, achieved an mAP@0.5 of 70.7 with 11.1 M parameters and 28.7 GFLOPs. This sets a benchmark for subsequent technological enhancements. Notably, the inclusion of Wise-IoU did not increase the parameter count or computational cost, yet improved model accuracy without additional overhead. Therefore, Wise-IoU was incorporated as a foundational component in the ablation study. Following the inclusion of MGEFA, the mAP@0.5 increased to 71.2, indicating MGEFA’s significant impact on enhancing feature extraction and fusion capabilities while maintaining the model’s lightweight nature due to minimal changes in the parameter count and computational complexity. This improvement underscores the effectiveness of MGEFA in capturing complex features, which is crucial in scenarios where objects have intricate textures or are partially occluded. However, we observed that in some cases, the increase in mAP@0.5 was more pronounced for certain object classes, suggesting that MGEFA may benefit from further tuning to generalize across all categories uniformly.

The inclusion of the P6 layer significantly enhanced model performance; YOLOv8s-P6 reached an mAP@0.5 of 73.0. This layer plays a crucial role in optimizing the model’s ability to handle multi-scale objects and complex backgrounds. Although the parameter count increased to 17.8 M, there was no significant increase in GFLOPs, indicating that computational load remained similar and suggesting potential in maintaining inference speed. However, the increased parameter count could pose challenges for deployment on resource-constrained devices. To address this, we are exploring model compression techniques such as pruning and quantization in future work to reduce the model size without sacrificing accuracy. The introduction of LSKA improved the mAP@0.5 to 71.6, significantly aiding in the model’s ability to capture finer details by enhancing spatial information processing efficiency. Despite a slight increase in GFLOPs, overall computational efficiency was optimized due to LSKA’s efficient processing mechanisms. This suggests that LSKA effectively balances the trade-off between computational cost and detection accuracy. In particular, LSKA enhances the model’s attention mechanism, allowing it to focus on relevant features while ignoring irrelevant background noise.

Evaluating the combination of different technologies, the model incorporating both P6 layer and MGEFA (YOLOv8s-P6-MGEFA) achieved an mAP@0.5 of 72.8, showing that this combination could enhance model performance to some extent. Interestingly, the performance gain from combining P6 and MGEFA was slightly less than the sum of their individual improvements, indicating some overlap in the functionalities they provide. This suggests that while both components enhance multi-scale feature representation, their combined effect may reach a saturation point. The model combining a P6 layer and LSKA (YOLOv8s-P6-LSKA) achieved an mAP@0.5 of 73.2, indicating a better performance improvement. The components of this combination appear to complement each other well, as P6 enhances multi-scale feature extraction while LSKA improves spatial information processing. The synergy between these components results in better detection of objects in complex scenarios. Moreover, the model that integrated LSKA and MGEFA (YOLOv8s-LSKA-MGEFA) reached an mAP@0.5 of 71.7, demonstrating a synergistic effect when these technologies were applied together. However, the performance improvement was less than when P6 was involved, highlighting the crucial role of the P6 layer in handling multi-scale objects. This indicates that while LSKA and MGEFA enhance certain aspects of the model, they may not fully compensate for the benefits provided by the P6 layer.

Most notably, the comprehensive application of the P6 layer, LSKA, and MGEFA in the model (YOLOv8s-P6-LSKA-MGEFA) resulted in the highest mAP@0.5 of 73.4 among all combinations. Despite an increase in the parameter count to 19.0 M, the model maintained high computational efficiency with 28.0 GFLOPs, which is slightly lower than the baseline YOLOv8s model’s 28.7 GFLOPs. This illustrates that the integrated application of these technologies not only enhanced the detection accuracy but also maintained high computational efficiency. This result demonstrates the effectiveness of combining these components to address different challenges in object detection. The P6 layer improves multi-scale feature representation, MGEFA enhances feature extraction and fusion, and LSKA optimizes spatial information processing. Together, they provide a robust framework for detecting objects in complex environments.

Despite these improvements, we acknowledge certain limitations in our study. One challenge we encountered is that although the overall floating-point operations did not increase with the addition of the P6 layer, the model size grew, which may limit its deployment on devices with strict memory constraints. Additionally, while our model performs well on the tested dataset, its generalization to other datasets or real-world applications with different characteristics remains to be validated.

**Figure 9:**
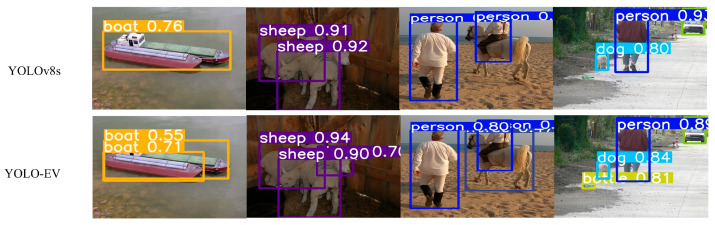
Comparison chart of experimental results between the improved model YOLO-EV and YOLOv8s.

Figure 9 compares the actual detection capabilities of the YOLOv8s and YOLO-EV models, highlighting that YOLO-EV effectively addresses issues present in YOLOv8s in complex environments, such as occluded and small objects. In practical applications, this enhanced detection capability translates to better performance in tasks such as autonomous driving, surveillance, and robotics, where accurately detecting objects under varying conditions is critical.

To validate the effectiveness of the improved methods on other scale YOLOv8 models, we applied the four proposed enhancement techniques (MGEFA, P6 layer, WIoU, and LSKA) to the smaller-scale YOLOv8n and the larger-scale YOLOv8m models, resulting in the enhanced models YOLOv8n-EV, YOLOv8s-EV, and YOLOv8m-EV. In this context, we refer to YOLOv8s-EV as YOLO-EV, as it strikes a balance between model size and the speed and accuracy of detection. The experimental results are listed in Table 3. By conducting experiments on models of different sizes, it has been demonstrated that these improvement methods are equally effective across all models. Specifically, from YOLOv8n to YOLOv8m, the enhanced models showed significant improvements in performance metrics such as mAP, achieving the effect of expanding the model’s “field of view.” This indicates that our improvement methods have good versatility, being applicable not only to the YOLOv8s model but also to other models in the YOLOv8 series. These experimental outcomes provide guidance for applying our improvement methods to more model architectures, proving that employing techniques like MGEFA, P6 layers, WIoU, and LSKA can effectively enhance the detection performance and generalization ability of models across different scales.

This ablation study thoroughly demonstrates the effectiveness and applicability of MGEFA, P6 layer, WIoU, and LSKA technologies in enhancing the detection accuracy of the YOLO-EV model. Through the optimization and combination of these technologies, the YOLO-EV model’s ability to detect targets in complex scenarios has been significantly improved, providing robust support for further model enhancements and practical applications. Our findings suggest that future research could explore the integration of additional techniques such as attention mechanisms [22] or transformer-based [44] modules to further enhance performance. Moreover, investigating the model’s performance on different datasets would provide insights into its generalization capabilities.

This study also offers a set of guidelines for optimizing object detection models, suggesting that the technologies discussed herein can be applied to other algorithms to expand the model’s capabilities. For instance, integrating MGEFA or LSKA into other models like Faster R-CNN or SSD could potentially yield similar improvements. By sharing our experiences and insights, we aim to contribute to the broader research community’s efforts in advancing object detection technologies.

## 4. Application

The application of object detection technology in the agricultural sector is increasingly gaining attention, particularly in precision agriculture, where real-time detection of weeds plays a crucial role in enhancing crop yields and reducing the use of chemical herbicides. Advanced computer vision techniques enable the automatic detection and classification of weeds in fields, thus effectively supporting intelligent agricultural decision-making and operations.

Weed detection faces numerous challenges. First, the high density of weed environments increases the difficulty of distinguishing weeds from crops due to their similarity. Secondly, the small size and variability of weeds present a technical challenge in accurately identifying weed locations and types across extensive fields. Additionally, the diversity of weeds significantly increases the complexity of model training. Moreover, varying environmental conditions such as changes in lighting, shadows, and weather can adversely affect image quality, further complicating the detection process. This paper employs the YOLO-EV model, tested and validated on specific agricultural datasets, namely, CottonWeedDet12 (CWD12) and CropWeed. Owing to its outstanding performance in handling high-density scenes with small objects, the YOLO-EV model is particularly well-suited for weed detection applications. Test results indicate that the YOLO-EV model effectively differentiates between weeds and crops, enhancing the accuracy and real-time capabilities of weed detection. The CWD12 and CropWeed datasets, specifically designed for agricultural weed detection, include a large number of images of various types of weeds and crops. All images are maintained at a uniform size during the training process to ensure consistency in model training and maximize the effectiveness of model training. By successfully applying YOLO-EV to weed detection, we demonstrate its potential for broader applications in precision agriculture, such as pest detection [45], crop health monitoring [46], and yield estimation [47]. The ability to accurately detect and classify objects in real-time can significantly improve decision-making processes, reduce labor costs, and increase overall agricultural productivity.

### 4.1. Detection of Cotton Field Weeds Using the CWD12 Dataset

The advancement of weed detection technology provides critical tools for precision agriculture, especially in the realization of automated and intelligent agricultural management. YOLO-WDNet [48], a lightweight and efficient model developed specifically for cotton field weed detection, employs advanced algorithms and structural optimizations to significantly enhance detection performance and efficiency, and its effectiveness has been validated in practical applications. Another model, YOLOv4-weeds [49], optimized for weed detection in carrot fields, utilizes MobileNetV3-Small as its backbone network, significantly improving detection efficiency and accuracy, making it particularly suitable for embedded devices and effectively supporting precision agriculture applications. Additionally, the performance of the YOLOv5 model in automatically identifying crops and weeds from drone imagery has been evaluated [50], providing key insights for implementing deep learning in precision agriculture.

A review of the existing literature reveals that despite various technologies proposed for identifying and classifying weeds, numerous challenges remain in practical applications. Weed detection in actual agricultural fields faces many challenges, including the visual similarity between weeds and crops, as well as the variability and occlusion of weeds, which often lead to insufficient detection accuracy and misidentification. Moreover, the complexity of agricultural scenes, such as varying lighting conditions and weather changes, greatly increases the difficulty in developing weed recognition technologies. This study employs the advanced YOLO-EV model, validated on the CWD12 dataset comprising 4518 training images and 1130 validation images. The CWD12 dataset, designed specifically for weed detection, contains a large number of images of weeds and crops in agricultural fields. The YOLO-EV model’s exceptional capabilities in handling complex scenes and small targets make it an ideal choice for weed detection. By incorporating techniques such as MGEFA and LSKA, YOLO-EV enhances feature extraction and spatial information processing, which are crucial for distinguishing between visually similar objects in cluttered backgrounds.

**Figure 10:**
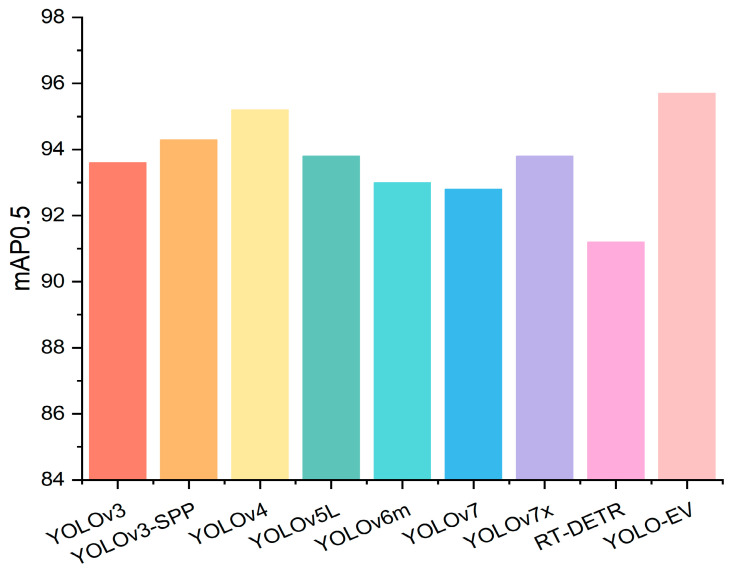
Comparison of experimental results for the CWD12 dataset.

As shown in Table 4, the YOLO-EV model not only outperforms other models in terms of mAP but also exhibits lower computational complexity, demonstrating its potential for application in complex agricultural environments. Specifically, YOLO-EV achieved an mAP@0.5 of 95.7, outperforming models like YOLOv7 and RT-DETR. This performance gain is significant for practical applications where even small improvements in detection accuracy can lead to substantial economic benefits by reducing crop losses due to weeds. Figure 10 presents a bar chart of mAP@0.5, highlighting the outstanding performance of the YOLO-EV model.

**Figure 11:**
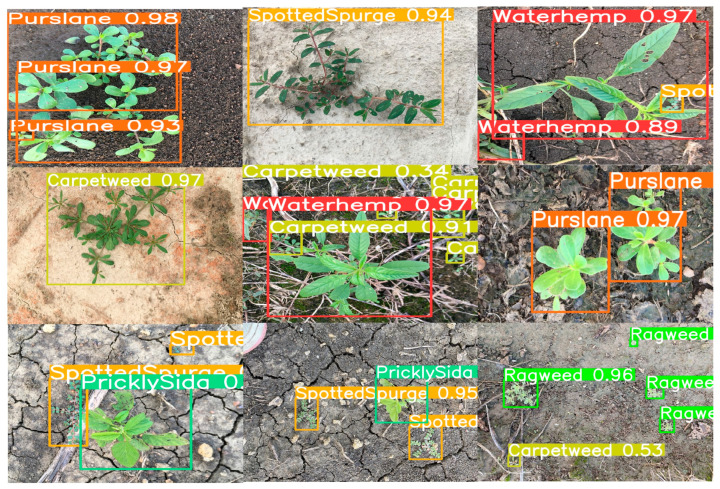
Display of experimental results for the CWD12 dataset.

Figure 11 illustrates the experimental results of the YOLO-EV model, which effectively distinguishes between weeds and crops. It is observed that the YOLO-EV model excels in identifying small weeds in complex scenes and can effectively handle occlusions, as demonstrated in the test results on the validation set. Furthermore, with a frame rate of 104 FPS, it meets the requirements for real-time detection. This high processing speed enables its deployment on agricultural machinery equipped with cameras for on-the-fly weed detection and spraying, thereby reducing the need for manual intervention and enabling precision herbicide application.

Despite these promising results, challenges remain. The model’s performance under varying environmental conditions, such as extreme lighting or weather changes, requires further evaluation. Additionally, the robustness of the model across different growth stages of crops and weeds is an area for future research.

### 4.2. Detection of Sesame Weeds Using the CropWeed Dataset

**Figure 12:**
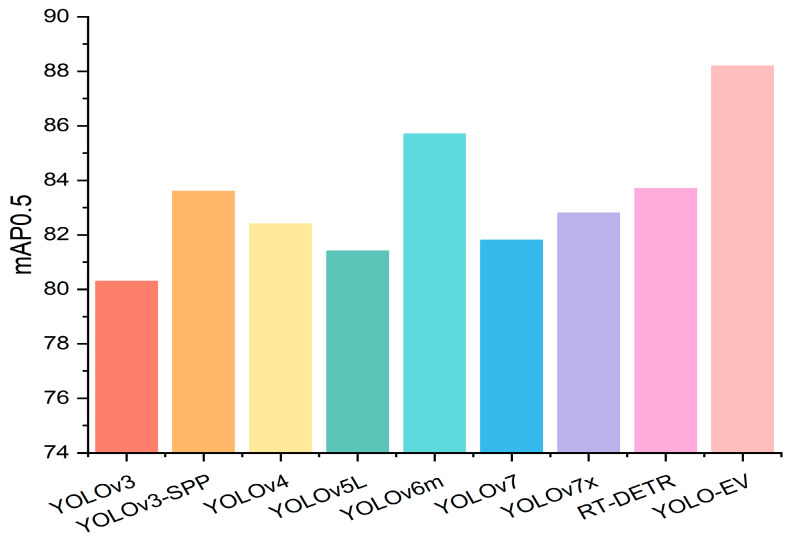
Comparison of experimental results for the CropWeed dataset.

Sesame is an important oilseed crop but is highly sensitive to weed competition in its early growth stages, necessitating effective weed control methods. Traditional machine vision techniques perform well in weed detection; however, in complex sesame fields, the similarity in morphology between crops and weeds may limit the feature extraction capabilities and accuracy of the model. To address this issue, the YOLO-EV model proposed in this paper enhances the detection speed while improving the accuracy of weed identification, adapting to the needs of complex field environments. By leveraging advanced feature extraction and attention mechanisms, the model effectively differentiates between sesame plants and weeds, even in challenging conditions. The model’s performance in complex agricultural settings was validated through experiments on the CropWeed dataset, which included 1040 training images and 260 validation images, ensuring the model’s generalization ability and accuracy under various conditions. Table 5 shows the experimental results, indicating that the YOLO-EV achieves high mAP values while maintaining low GFLOPs, demonstrating its excellent performance in both recognition accuracy and computational efficiency. Specifically, YOLO-EV achieved an mAP@0.5 of 88.2. This improvement in accuracy is critical for applications where precise weed identification directly impacts crop yield and quality. Figure 12 presents a bar chart of mAP@0.5, highlighting the superior results achieved by the YOLO-EV model.

**Figure 13:**
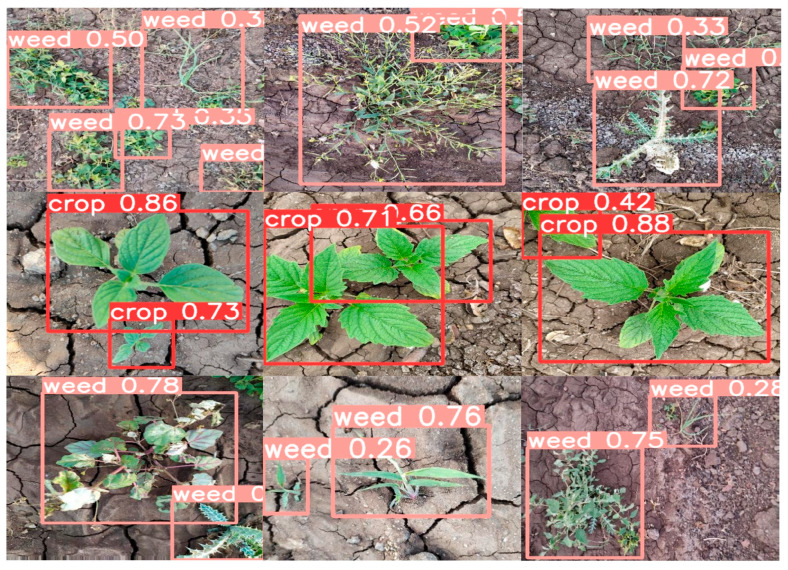
Display of experimental results for the CropWeed dataset.

Figure 13 demonstrates the application of the YOLO-EV model on the CropWeed dataset, indicating that the model effectively distinguishes between weeds and sesame. It shows excellent performance in handling small weeds and complex backgrounds. With a frame rate of 106 FPS, it meets the requirements for real-time detection. The model’s high accuracy and rapid processing capabilities make it a powerful tool for weed management in practical agricultural applications. This enables real-time monitoring and intervention, such as automated weed removal systems that can operate efficiently across large agricultural fields.

In practical deployment, challenges such as the need for high-quality image capture equipment and potential difficulties in maintaining model performance over time due to changes in environmental conditions or weed populations should be considered. Future work may involve integrating the model with other techniques to continuously update and improve performance.

## 5. Conclusions

In this paper, we introduced an advanced object detection model, termed the efficient optimized YOLOv8 model with extended vision (YOLO-EV), which is engineered to address the challenges of weed detection in complex agricultural settings. By incorporating innovative technologies such as multi-branch group-enhanced fusion attention (MGEFA), large-scale kernel attention (LSKA), and the Wise-IoU loss function, we enhanced the YOLOv8 architecture to significantly improve its capability in handling spatial information and multi-scale object detection, leading to superior performance in complex agricultural environments. Extensive experimental results on multiple datasets, including CottonWeedDet12 and CropWeed, demonstrate that YOLO-EV outperforms existing models in terms of accuracy and efficiency, confirming its effectiveness. Specifically, on the CottonWeedDet12 and CropWeed agricultural datasets, YOLO-EV achieved mAP@0.5 scores of 95.2 and 88.2, respectively, not only achieving high accuracy but also effectively managing the detection of occluded and small-sized targets, which are common challenges in agricultural object detection. These findings validate not only the efficiency and practical utility of YOLO-EV in real-world agricultural applications but also its potential to significantly enhance precision agriculture practices by enabling accurate and real-time weed detection. Comparisons with the traditional YOLOv8 and other state-of-the-art models show that YOLO-EV excels in accuracy, computational efficiency, and resource consumption. Additionally, the innovative design of the model, including the integration of MGEFA and LSKA modules, sets a new precedent for object detection models, providing a framework that can be extended to other domains facing similar challenges, thereby opening new avenues and possibilities for future object detection tasks in more complex scenarios. In summary, the development of the YOLO-EV model represents a significant advancement in the field of object detection, successfully addressing specific challenges in complex agricultural environments, such as detecting small, occluded, and densely clustered weeds, thereby pushing the boundaries of object detection technology and making substantial contributions to the advancement of precision agriculture. Future work will focus on further optimizing the model structure to enhance its adaptability and robustness across a broader range of application scenarios, including exploring transfer learning techniques to apply YOLO-EV to different crops and environmental conditions and integrating the model into autonomous agricultural machinery for real-time weed management.

## Figures and Tables

**Table 1:** Configuration of the experimental environment.

Hardware or Software Platform	Model Identification or Name	Parameters or Version
CPU	Intel(R USA) Xeon Gold 6330 (Intel Corporation, Santa Clara, CA, USA)	Frequency: 2.00 GHz
GPU	NVIDIA GeForce RTX 3090Ti (NVIDIA, Santa Clara, CA, USA)	Video memory: 24
Computer system	Linux	RAM: 80 GB
Deep learning framework	PyTorch	1.13
Computational platform	CUDA	11.6
Integrated development environment	Vscode	2023.6
Programming language	Python	3.8

**Table 2:** Ablation study. The table demonstrates the performance comparison of the model under various configurations. In this table, we use “√” to indicate that a specific technological improvement has been integrated into the model, while “×” denotes that the technological improvement has not been applied. The table includes four technical fields: MGEFA, P6, LSKA, and WIoU, each corresponding to different technological improvements integrated into the model. This method of representation is designed to visually show the specific impact of different technology combinations on model performance.

Model	MGEFA	P6	LSKA	WIoU	Parameters (M)	GFLOPs	mAP@0.5
YOLOv8s	×	×	×	×	11.1	28.7	70.7
	×	×	×	√	11.1	28.7	71.1
	√	×	×	√	11.1	28.9	71.2
	×	√	×	√	17.8	28.6	73.0
	×	×	√	√	12.2	29.5	71.6
	√	√	×	√	17.9	28.9	72.8
	×	√	√	√	18.9	28.8	73.2
	√	×	√	√	12.2	19.6	71.7
	√	√	√	√	19.0	28.0	73.4

**Table 3:** Performance comparison and analysis of different scale YOLOv8 models with their enhanced versions.

Model	Parameters (M)	GFLOPs	mAP@0.5
YOLOv8n	3.0	8.2	67.7
YOLOv8n-EV	5.1	8.3	69.8
YOLOv8s	11.1	28.7	70.7
YOLOv8s-EV (YOLO-EV)	19.0	28.0	73.4
YOLOv8m	25.8	79.1	73.6
YOLOv8m-EV	46.5	83.6	75.3

**Table 4:** Experimental results on the CWD12 dataset.

Model	Parameters (M)	GFLOPs	mAP@0.5	mAP@0.5:0.95
YOLOv3	62	155	95.3	87.0
YOLOv3-SPP	63	63	94.3	87.5
YOLOv4	64	143	95.2	89.5
YOLOv5L	46	108	93.8	87.7
YOLOv6m	53	123	93.0	88.1
YOLOv7	37	105	92.8	86.5
YOLOv7x	71	189	93.8	89.1
RT-DETR	21	58	91.2	85.0
YOLOv8s	11	29	94.5	88.8
YOLO-EV	19	29	95.7	89.8

**Table 5:** Experimental results on the CropWeed dataset.

Model	Parameters (M)	GFLOPs	mAP@0.5	mAP@0.5:0.95
YOLOv3	62	155	80.3	50.4
YOLOv3-SPP	63	63	83.6	56.3
YOLOv4	64	143	82.4	56.9
YOLOv5L	46	108	81.4	51.4
YOLOv6m	53	123	85.7	59.4
YOLOv7	37	105	81.8	49.1
YOLOv7x	71	189	82.8	52.5
RT-DETR	21	58	83.7	57.6
YOLOv8s	11	29	87.2	60.1
YOLO-EV	19	29	88.2	60.4

## Data Availability

The data comes from a public dataset. It is available at http://host.robots.ox.ac.uk/pascal/VOC/voc2012 (accessed on 10 September 2023); https://zenodo.org/records/7535814 (accessed on 12 September 2023); https://www.kaggle.com/datasets/ravirajsinh45/crop-and-weed-detection-data-with-bounding-boxes (accessed on 12 May 2024). Additionally, once the related laboratory work is completed, the code will be made publicly available at https://github.com/Y2617/YOLO-EV (accessed on 12 May 2024).
